# A Comprehensive *In Silico* Analysis on the Structural and Functional Impact of SNPs in the Congenital Heart Defects Associated with NKX2-5 Gene—A Molecular Dynamic Simulation Approach

**DOI:** 10.1371/journal.pone.0153999

**Published:** 2016-05-06

**Authors:** Firoz Abdul Samad, Bandar A. Suliman, Syed Hussain Basha, Thamilarasan Manivasagam, Musthafa Mohamed Essa

**Affiliations:** 1 Center for Genetics and Inherited Diseases, Taibah University, Madinah, Kingdom of Saudi Arabia; 2 Molecular Biomedicine Program, Research Center, King Faisal Specialist Hospital & Research Center, Riyadh, Kingdom of Saudi Arabia; 3 Innovative Informatica Technologies, HIG, HUDA, Mayuri Nagar, Miyapur, Hyderabad, 500 049, India; 4 Department of Biochemistry and Biotechnology, Annamalai University, Annamalai Nagar, Tamilnadu, India; 5 Department of Food Science and Nutrition, College of Agricultural and Marine Sciences, Sultan Qaboos University, Muscat, Oman; 6 Aging and Dementia Research Group, Sultan Qaboos University, Muscat, Oman; Cincinnati Children's Hospital Medical Center, UNITED STATES

## Abstract

Congenital heart defects (CHD) presented as structural defects in the heart and blood vessels during birth contribute an important cause of childhood morbidity and mortality worldwide. Many Single nucletotide polymorphisms (SNPs) in different genes have been associated with various types of congenital heart defects. NKX 2–5 gene is one among them, which encodes a homeobox-containing transcription factor that plays a crucial role during the initial phases of heart formation and development. Mutations in this gene could cause different types of congenital heart defects, including Atrial septal defect (ASD), Atrial ventricular block (AVB), Tetralogy of fallot and ventricular septal defect. This highlights the importance of studying the impact of different SNPs found within this gene that might cause structural and functional modification of its encoded protein. In this study, we retrieved SNPs from the database (dbSNP), followed by identification of potentially deleterious Non-synonymous single nucleotide polymorphisms (nsSNPs) and prediction of their effect on proteins by computational screening using SIFT and Polyphen. Furthermore, we have carried out molecular dynamic simulation (MDS) in order to uncover the SNPs that would cause the most structural damage to the protein altering its biological function. The most important SNP that was found using our approach was rs137852685 R161P, which was predicted to cause the most damage to the structural features of the protein. Mapping nsSNPs in genes such as NKX 2–5 would provide valuable information about individuals carrying these polymorphisms, where such variations could be used as diagnostic markers.

## 1. Introduction

Single nucleotide polymorphisms (SNPs) are the most common genetic variations in any population; they occur when a single nucleotide in the genome (A, T, C or G) is altered [1]. Even though many SNP's have no effect on the biological functions of the cell, some can predispose people to certain diseases, influence their immunological response to drugs and can be considered as biomarkers for disease susceptibility [2]. Importantly, nsSNPs result in changes to the amino acid sequence of proteins and have been reported to be responsible for about 50% of all known genetic variations that are linked to inherited diseases [3]. On the other hand, coding synonymous (sSNPs) and those occuring seen outside gene coding or promoter regions may also influence transcription factor binding and gene expression [4, 5].

Even though the influence of genetics on susceptibility to cardiovascular diseases is well documented, delineation of the complete spectrum of the risk alleles was not achieved previously until the development of Next Generation Sequencing Techniques [6]. The recent advancement in high throughput sequencing has increased the rate at which DNA sequence variations are identified and subsequently deposited in genetic databases [7]. Moreover, because of the availability of such sequencing data from many databases, researchers have turned to bioinformatics tools to exploit these data and try to annotate and extract useful clinical information hidden within.

There is a great need for an effective and efficient method to filter out pathogenic and deleterious SNPs from the readily available pool of variant data, and to further explore the impact of those selected SNPs at the molecular level. Bioinformatics prediction tools can be used in a cost efficient manner for prioritizing SNPs of likely functional importance, enabling an investigation of the structural basis of disease-causing mutations likely to contribute to an individual's disease susceptibility [8–10]. Also, it is important to note that the success of association studies always depends on how a research group chooses a set of SNPs to be investigated [11, 12]. Without a detailed *In silico* analysis of SNPs to be screened, based on the functional importance, a large number of samples might be needed to identify association at an acceptable level of statistical significance [13, 14]. A comprehensive analysis of the functional and structural impact of SNPs in a gene will not only be supportive but also facilitate the discrimination between true associations and false positives as reported recently [8–10].

In recent years, there has been a considerable interest to study the genetic determinants of Congenital heart defects (CHD) as this was reported to be an important cause of childhood morbidity and mortality worldwide [6, 15–17]. The American heart association estimates that about 9 children out of 1000 are born with CHD [18]. Even though significant progress has been achieved in diagnostic and therapeutic strategies, the etiology of CHD is not well understood. But recent advancements in sequencing techniques have led to increasing evidences implicating a stronger role of single gene defect associated with various kinds of CHDs (16, 17). NKX 2–5 is an important gene that has been linked to CHD [19]. This gene encodes a homeobox-containing transcription factor which regulates tissue-specific gene expression involved in early heart formation and development. Mutations in this gene can cause different forms of congenital heart defects, including Atrial septal defect (ASD), Atrial ventricular block (AVB), Tetralogy of fallot (TF), Ventricular septal defect (VSD) [20]. Although several studies have been reported about associated SNPs in the NKX2-5 gene [21, 22], a molecular dynamics simulation analysis has not yet been performed to gain insight in to the impact of nsSNPs on the gene’s structural integrity.

The correlation between MDS analysis and experimental work is well established in various independent studies elsewhere [23, 24]. On the other hand, Kumar et.al, [25–27], Doss et.al, [28–29] and Rajendran et.al, [30–31] have recently demonstrated that computational SNP predictions are helpful in combination with MDS studies in finding out the most significant disease causing mutation, out of a pool of SNPs having predictable correlations with the wet lab experiments. Thus, MDS analysis was presumed to generate detailed information on the structural changes including residue fluctuations and conformational changes of protein resulting from a pathogenic mutation in perfect agreement with experimental methods. In the present study, a special focus has been given on the MDS analysis of potentially pathogenic SNPs towards revealing 1) the structural impact of the following SNP's- rs72554027 (F145S), rs397516909 (S146W), rs201582515 (V150I), rs137852685 (R161P), rs104893900 (T178M), rs3729938 (S179C), rs72554028 (Q181H) rs137852686 (K183E) and rs104893906 (R190C) 2) whether these mutations are causing any structural or conformational changes in the proteins, with reference to the wild type.

In summary, our work aimed to identify pathogenic variants and to find out which associated SNPs are causing the most damage to the structural features of the NKX2-5 protein, thus negatively affecting its biological functions.

## 2. Results and Discussion

### 2.1 Selection of SNP data set from db SNP

The dbSNP database reported a total of 1345 SNPs for the NKX2-5 gene, out of which 252 were found to be Human (active) SNPs which included: 65 coding nsSNPs, 29 coding synonymous, 78 in the mRNA 3' UTR region, 16 in the mRNA 5'UTR region and 64 in the intronic region (Table 1). It was noted that the vast majority of SNPs of this gene fall in the coding region, and the number of nsSNPs were higher than all the other types of SNPs. W this observation was different from the usual distribution of SNPs reported in many other genes (8, 31). Since non-synonymous SNPs could alter the encoded amino acid and are likely to be disease causing, we selected them for further analysis.

**Table 1:** Distribution of NKX2-5 nSNPs, sSNPs), 3'UTR SNPs, 5' UTR SNPs and intronic SNPs.

S.No	Type of SNP	No.of SNPs
1.	nsSNPs	65
2.	sSNPs	29
3.	mRNA 3' UTR	78
4.	mRNA 5'UTR	16
5.	Intronic region	64

### 2.2 Analysis of mutations in nsSNPs

Among the 65 nsSNPs selected for further analysis, 24 were predicted to be deleterious by the SIFT server with a tolerant index score less than or equal to 0.03 and the detailed result has been tabulated in S1 Table.

To further enhance the significance of SIFT predictions, the nsSNPs that were submitted to SIFT were also submitted to Polyphen 2.0 server. HDiv data set in Polyphen identified 52 SNPs as probably damaging with a high confident prediction score close ranges from 0.99 to 1. Whereas, HvarPred data set reported 9 SNPs as probably damaging with a prediction score between 0.99 to 1. Predicted HDiv damaging probabilities for the SNPs can be seen in S1 Table.

### 2.3 Effect of SNPs on polarity, hydrophobicity, structural stability and functionality of the protein

Each amino acid has its own unique properties, such as molecular weight, size, polarity/charge and hydrophobicity values. In view of this fact, we have studied the effect of SNPs on the protein structure, but paid more attention to its polarity and hydrophobicity, since they are the major contributors for the protein’s structure and functionality. The results of the detailed analysis for effect of mutations on protein’s polarity and hydrophobicity are presented in Table 2. From the analysis it was revealed that, among the nine presently available mutations, the majority of mutations have shown polarity changes with the exception of V150I. On the other hand, potentially hydrogen bond forming residues, serine and threonine, were found to be mutated with non-hydrogen bond forming residues, whereas in some cases, non-hydrogen bond forming residues have been found to be mutated with hydrogen bond forming residues serine, cysteine and methionine.

**Table 2:** Details of polarity and hydrophobicity/hydrophilicity of the reported SNP’s for NKX 2.5 along with their effect on mutated proteins.

S/N	Mutation	Polarity	Change in polarity due to mutation	Hydrophobicity/ Hydrophilicity	Change in Hydrophobicity/ Hydrophilicity due to mutation
1.	F145S	Neutral to Polar	√	Hydrophobic to Hydrophilic	√
2.	S146W	Neutral Polar to Nonpolar	√	Hydrophilic to Hydrophilic	x
3.	V150I	Neutral Non-polar to Neutral Non-polar	x	Hydrophobic to Hydrophobic	x
4.	R161P	Polar to Nonpolar	√	Hydrophilic to Hydrophobic	√
5.	T178M	Polar to Nonpolar	√	Hydrophilic to Hydrophilic	x
6.	S179C	Polar to Nonpolar	√	Hydrophilic to Hydrophilic	x
7.	Q181H	Neutral Polar to Basic Polar	√	Hydrophilic to Hydrophilic	x
8.	K183E	Basic Polar to Acidic polar	√	Hydrophilic to Hydrophilic	x
9.	R190C	Polar to Nonpolar	√	Hydrophilic to Hydrophilic	x

The above predictions were based on the standard evaluations that Glutamine (Q); Asparagine (N); Histidine (H); Serine (S); Threonine (T); Tyrosine (Y); Cysteine (C); Methionine (M); Tryptophan (W) were all potentially hydrogen bond-forming residues. Polarity changes in the protein, due to the above-mentioned mutations, might cause severe malfunctions in the protein with even minor changes in pH conditions. Moreover, the nine mutations that were mentioned above were found to be causing hydrophobicity changes in the protein along with mutations at F145S and R161P positions. Among these mutations, F145S was found to be altered from a hydrophobic residue to hydrophilic one, whereas R161Pwas observed to be converted from hydrophilic to hydrophobic residue are shown in Table 2.

### 2.4 MD simulations

In order to understand the conformational changes in the protein due to these mutations comparatively, we have carried out 10 nanoseconds of MDS for each protein. Various parameters have been analyzed throughout the simulation trajectory, especially Root mean square deviation (RMSD), Root mean square fluctuations (RMSF), energy parameters, total number of intra -molecular hydrogen bonds, radius of gyration and the secondary structure elements (SSE) of the protein with the time dependent function of MDS. All the statistically significant results of the simulations have been presented in the Table 3. Chemical timescale used in this present study is of enough for the side chain rearrangements in protein’s native state and to facilitate various conformations; on the other hand, recent studies demonstrated thatthe dynamic motions of a single protein molecule are self-similar and look the same, how long you look at them for, from picoseconds to hundreds of seconds [32].

**Table 3:** Statistical analysis for the MD simulations trajectory of wild and mutated NKX 2.5 proteins.

S.No	Protein	Energy (Kcal./mol)						Radius of Gyration		Intra molecular H-Bonds		RMSD	
		Total energy		VDW Energy		Coulomb’s energy							
		Range	Mean	Range	Mean	Range	Mean	Range	Mean	Range	Mean	Range	Mean
	WT	-3181 to -2009	-2676	-162 to -29	-98	-1594 to -1197	-1393	10.9 to 13.7	11.6	33 to 55	44	0 to 6.0	4.3
	F145S	-3193 to -2308	-2720	-169 to -32	-102	-1617 to -1213	-1434	11.5 to 14.2	12.4	30 to 54	44	0 to 6.1	3.0
	S146W	-3456 to -2293	-2834	-164 to -34	-106	-1623 to -1249	-1446	11.4 to 13.5	11.9	32 to 52	41	0 to 7.2	4.8
	V150I	-3213 to -2258	-2746	-150 to -38	-100	-1616 to -1224	-1427	11.3 to 13.2	11.9	30 to 54	42	0 to 5.4	3.1
	R161P	-3304 to -1994	-2716	-170 to -40	-105	-1530 to -1176	-1377	11.1 to 13.3	11.8	33 to 55	42	0 to 5.8	4.3
	T178M	-3245 to -2031	-2612	-170 to -36	-99	-1591 to -1219	-1397	11.4 to 13.1	12.0	30 to 55	42	0 to 6.6	4.5
	S179C	-3282 to -2097	-2684	-168 to -42	-107	-1581 to -1242	-1420	11.5 to 13.1	12.3	31 to 56	43	0 to 4.0	2.4
	Q181H	-3043 to -2096	-2549	-166 to -57	-111	-1562 to -1188	-1366	11.4 to 13.8	12.6	35 to 56	45	0 to 7.2	4.6
	K183E	-3049 to -2142	-2569	-171 to -37	-106	-1604 to -1277	-1440	11.1 to 13.4	11.9	32 to 59	43	0 to 5.5	3.1
	R190C	-3012 to -2034	-2554	-178 to -50	-115	-1540 to -1158	-1368	11.7 to 13.3	12.5	34 to 54	43	0 to 7.5	4.2

### 2.5 Protein structure conformational flexibility and stability analysis

**Fig 1:**
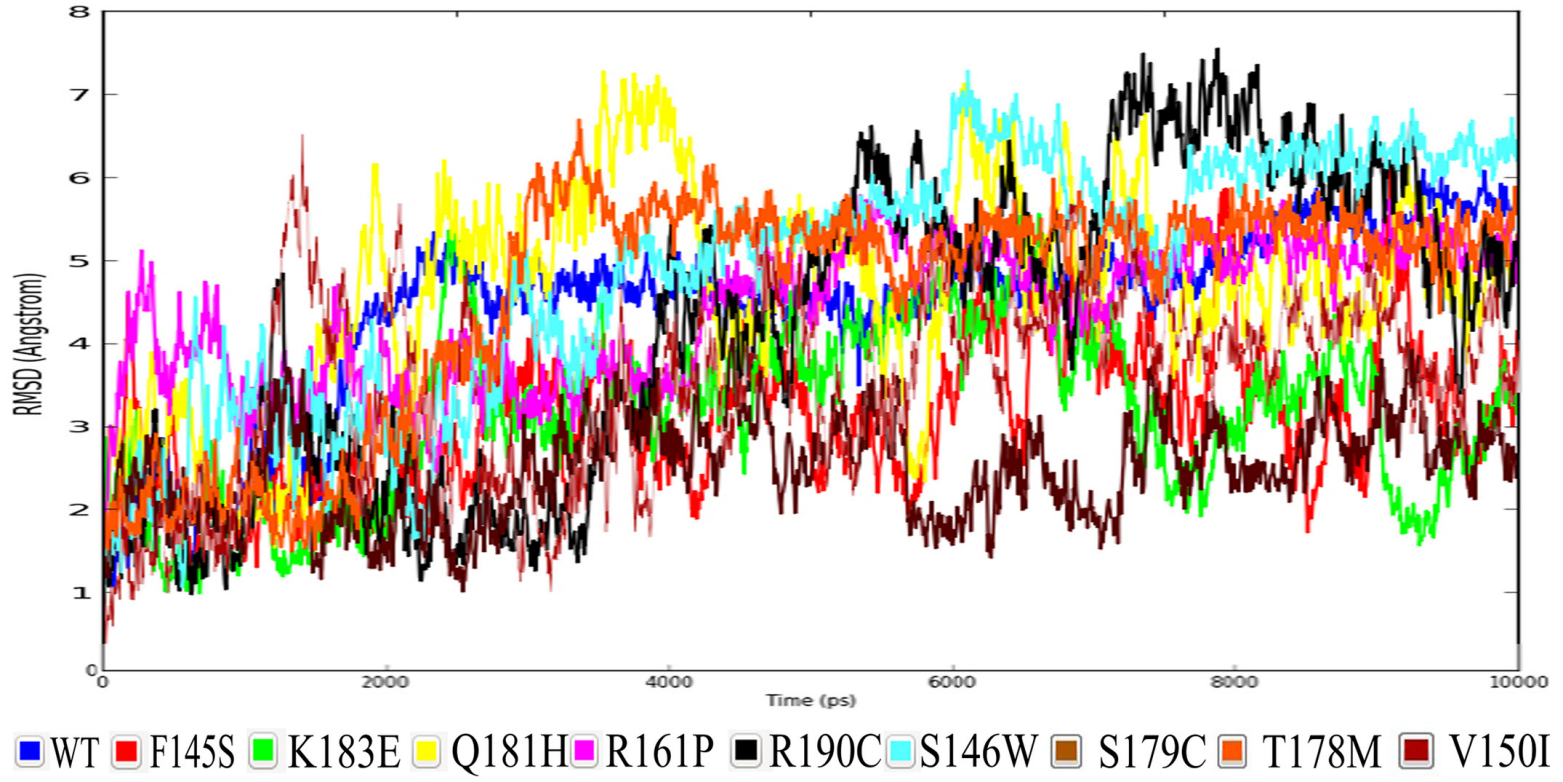
RMSD values of the native wild type protein along with those of the associated mutant proteins.

RMSD values of the wild type and mutant proteins were analyzed in order to understand the effect of the mutations on the protein structure. We calculated the RMSD for all protein backbones during the MDS with reference to its initial structure. Fig 1 shows that RMSD values from the mutant structures is quite unstable when compared to the wild type protein. However, the wild type protein was found to be stabilized at an RMSD value of around 5 Å, whereas most of the mutant protein RMSD values were lower than the wild type, but unstable with exception of S146W, T178M, R190C and T178M which were higher than the wild type protein’s RMSD at certain points. Fig 1 clearly demonstrates that the mutations have considerable destabilizing effects on protein structure.

**Fig 2:**
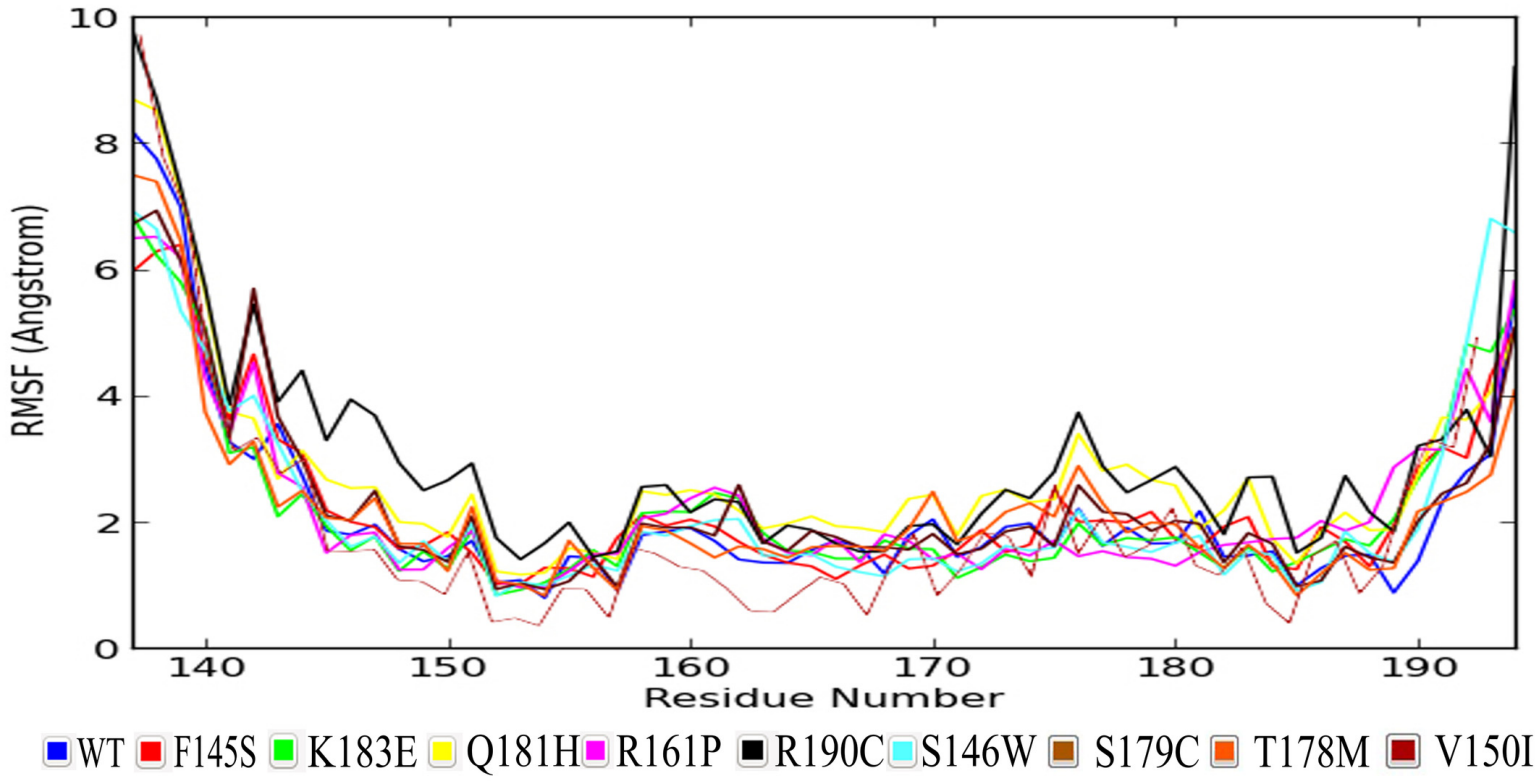
RMSF values of the native wild type protein along with those of the associated mutant protein.

We have also monitored the RMSF fluctuations of each residue in order to determine the mutation’s effect on the protein residues dynamic behavior. From Fig 2, it can be inferred that residue level fluctuations for R190C were quite high when compared with native and other mutations up to 4 Å, for residues located between 140 and 150 positions, while the next highest peak was observed approximately at the residue position at 175. Analysis of the fluctuations revealed that the greatest degree of flexibility was shown by the R190C mutant protein.

**Fig 3:**
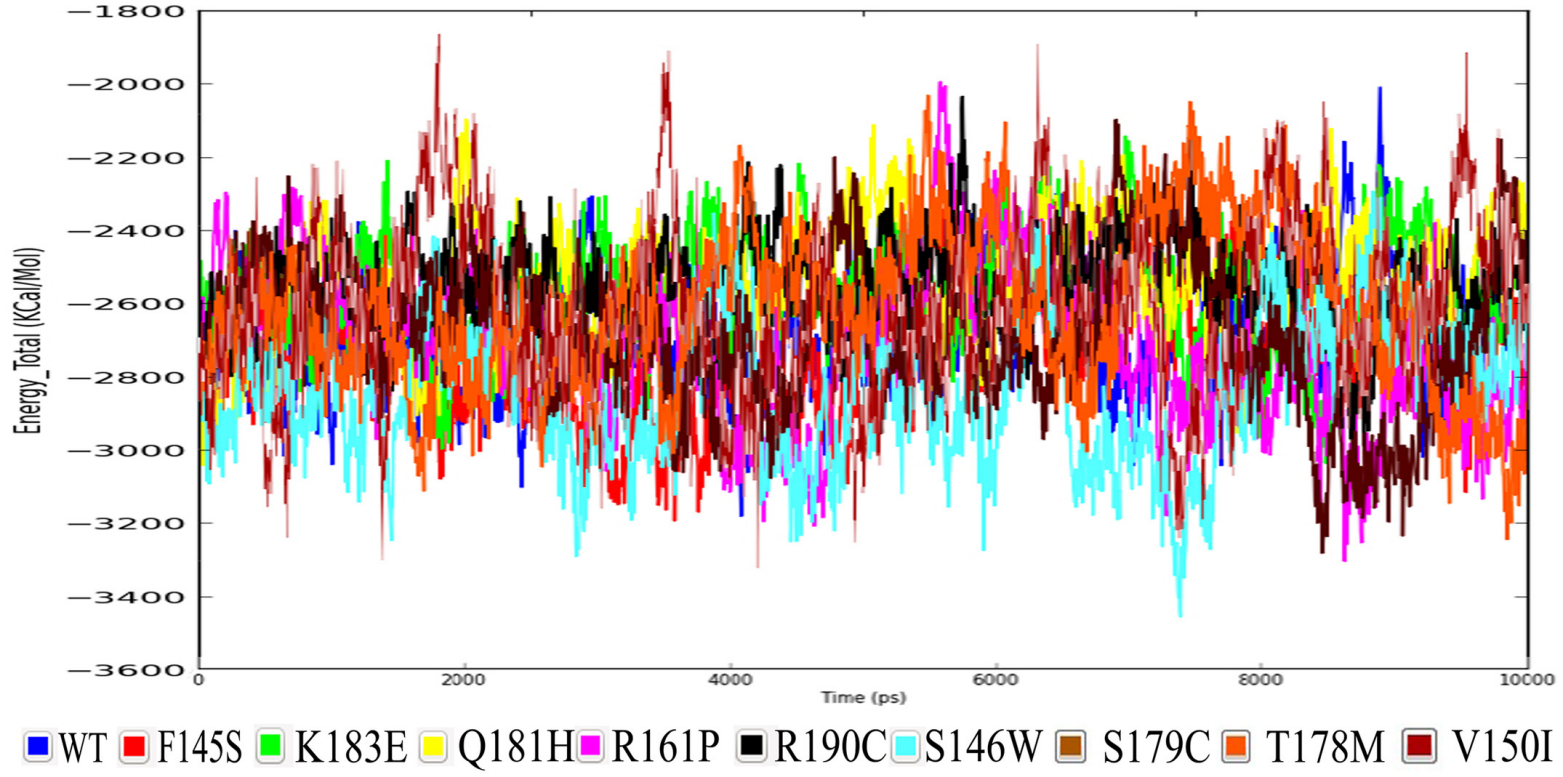
Total energy of the NKX 2.5 protein compared to that of each mutant protein.

When the energy parameters for the MD simulation trajectories of the wild type along with associated mutated proteins were analyzed, it was revealed that they were maintaining the total energy of the system in a range of -2834 and -2549Kcal/mol (Fig 3) whereas, for the wild type protein, -2676 Kcal/mol of energy was noted. Among the above mentioned energies,S146W mutated protein was showing the least possible minimized total energy of -2834 Kcal/mol, whereas the highest energy of -2549 Kcal/mol was found to be consumed by Q181H mutation. Also, as shown in Table 3, it is clear that as the mutation residue position increases, the total energy of the system increases as well, suggesting that the mutations occurring at the core of the protein structure would further minimize its overall energy, which is somehow contrary to the case of mutations occurring near the 3’ end of the protein.

When Vander Waal’s interaction contributions were calculated, it was found to be in a range of -98 and -115 Kcal/mol. The least energy of -115 Kcal/mol was observed to be for R190C mutation, whereas the highest -98 kcal/mol of energy was observed to be for the wild type protein. When Coulomb’s energy of the protein was analyzed, S146W was found to be maintaining the highest average of -1446 kcal/mol of energy and Q181H was found to be maintaining the least average of -1366 kcal/mol, whereas, the wild type protein was found to be maintaining an average of -1393 kcal/mol of energy.

**Fig 4:**
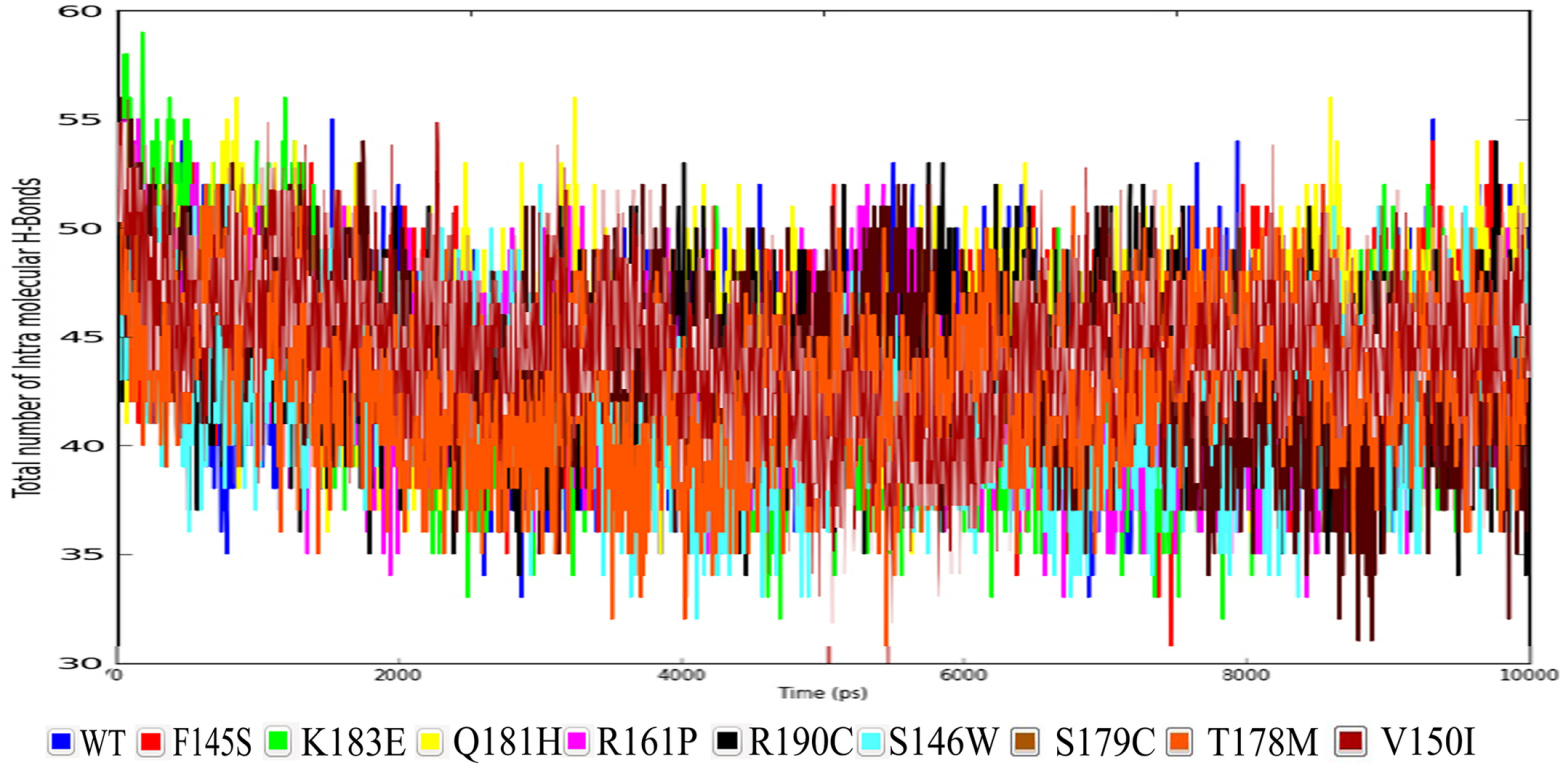
Total number of intra molecular hydrogen bond for the protein NKX 2.5 along with its reported mutations.

Next, we have analyzed the total number of intra-molecular hydrogen bonds present in the protein along with its reported mutations contributing for their stability (Fig 4). From Table 4 it can be inferred that the maximum number of 45 intra-molecular hydrogen bonds was observed for Q181H followed by 44 for wild type protein and the F145S mutation, followed by 43 for S179C, K183E and R190C mutations. The least number of 42 intra H-bonds were found for V150I, R161P and T178M mutations. This data suggests that V150I, R161P and T178M mutations have higher flexibility compared to other mutations and wild type protein.

**Table 4:** Details of Important SNP’s for NKX 2.5 along with their availability with presently available 3D structure of this protein.

S.No	Mutation	SNP	Availability in 3D structure
1.	K15I	rs387906773	Not available
2.	D16A	rs17052019	Not available
3.	E21Q	rs104893904	Not available
4.	Q22R	rs201442000	Not available
5.	R25C	rs28936670	Not available
6.	E32K	rs552617433	Not available
7.	A42P	rs113818864	Not available
8.	A55T	rs567939950	Not available
9.	A57P	rs549161381	Not available
10.	P59A	rs387906775	Not available
11.	A63V	rs530270916	Not available
12.	G74D	rs201362118	Not available
13.	C82S	rs150813574	Not available
14.	F86S	rs373807012	Not available
15.	P100A	rs550046293	Not available
16.	A112V	rs534163213	Not available
17.	A115V	rs529610517	Not available
18.	L116R	rs112167223	Not available
19.	A119S	rs137852684	Not available
20.	**F145S**	**rs72554027**	**Available**
21.	**S146W**	**rs397516909**	**Available**
22.	**V150I**	**rs201582515**	**Available**
23.	**R161P**	**rs137852685**	**Available**
24.	**T178M**	**rs104893900**	**Available**
25.	**S179C**	**rs3729938**	**Available**
26.	**Q181H**	**rs72554028**	**Available**
27.	**K183E**	**rs137852686**	**Available**
28.	**R190C**	**rs104893906**	**Available**
29.	P211L	rs3729754	Not available
30.	P212R	rs372282873	Not available
31.	R216C	rs104893905	Not available
32.	A219V	rs104893902	Not available
33.	P236H	rs397515399	Not available
34.	P257A	rs387906776	Not available
35.	Y259F	rs553883993	Not available
36.	P275T	rs368366482	Not available
37.	S279A	rs571382279	Not available
38.	P283Q	rs375086983	Not available
39.	F292L	rs538010963	Not available
40.	F295L	rs150581386	Not available
41.	G296D	rs373421818	Not available
42.	V297F	rs569535312	Not available
43.	G298E	rs549406766	Not available
44.	D299G	rs137852683	Not available
45.	A302E	rs371380388	Not available
46.	S311N	rs142368156	Not available
47.	G314A	rs200152391	Not available
48.	V315M	rs201249977	Not available
49.	R322P	rs376426882	Not available

**Fig 5:**
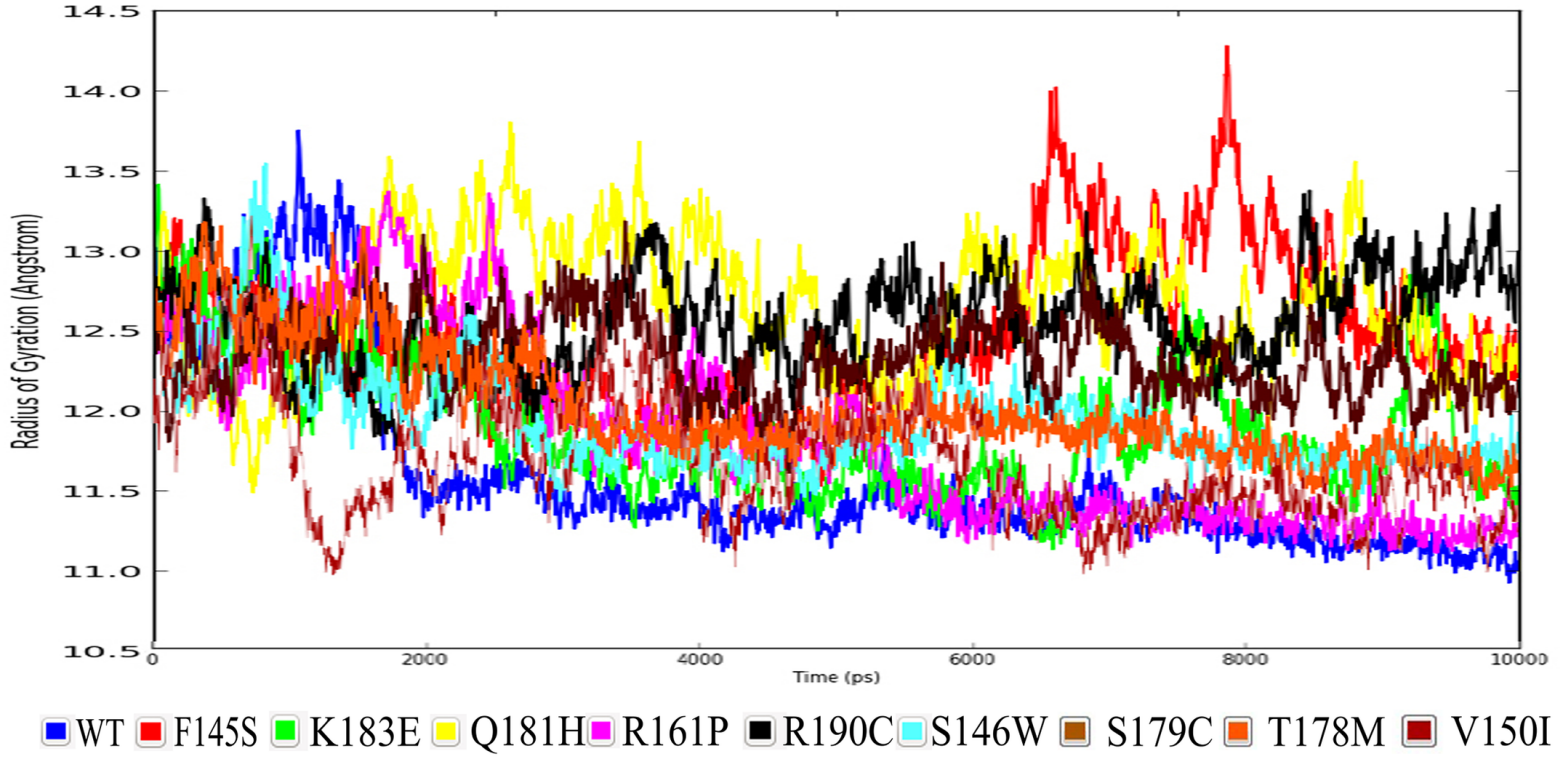
Radius of Gyration for the protein NKX 2.5 along with its associated mutations.

Finally, we have analyzed the radius of gyration (ROG) for the native wild type protein along with its associated mutations contributing to their compactness (Fig 5). From Table 3 it can be inferred that R190C mutation possessing protein has the least compactness of its structure with 12.5 Å when considering the statistical data, however R161P mutation has shown the highest compactness in the protein with 11.8 Å, whereas the wild type protein has shown to be highly compacted with 11.6 Å. These data suggests that all the mutations have caused structural destabilizing effects leading to the loss of protein compactness when compared to the data with wild type ROG.

**Fig 6:**
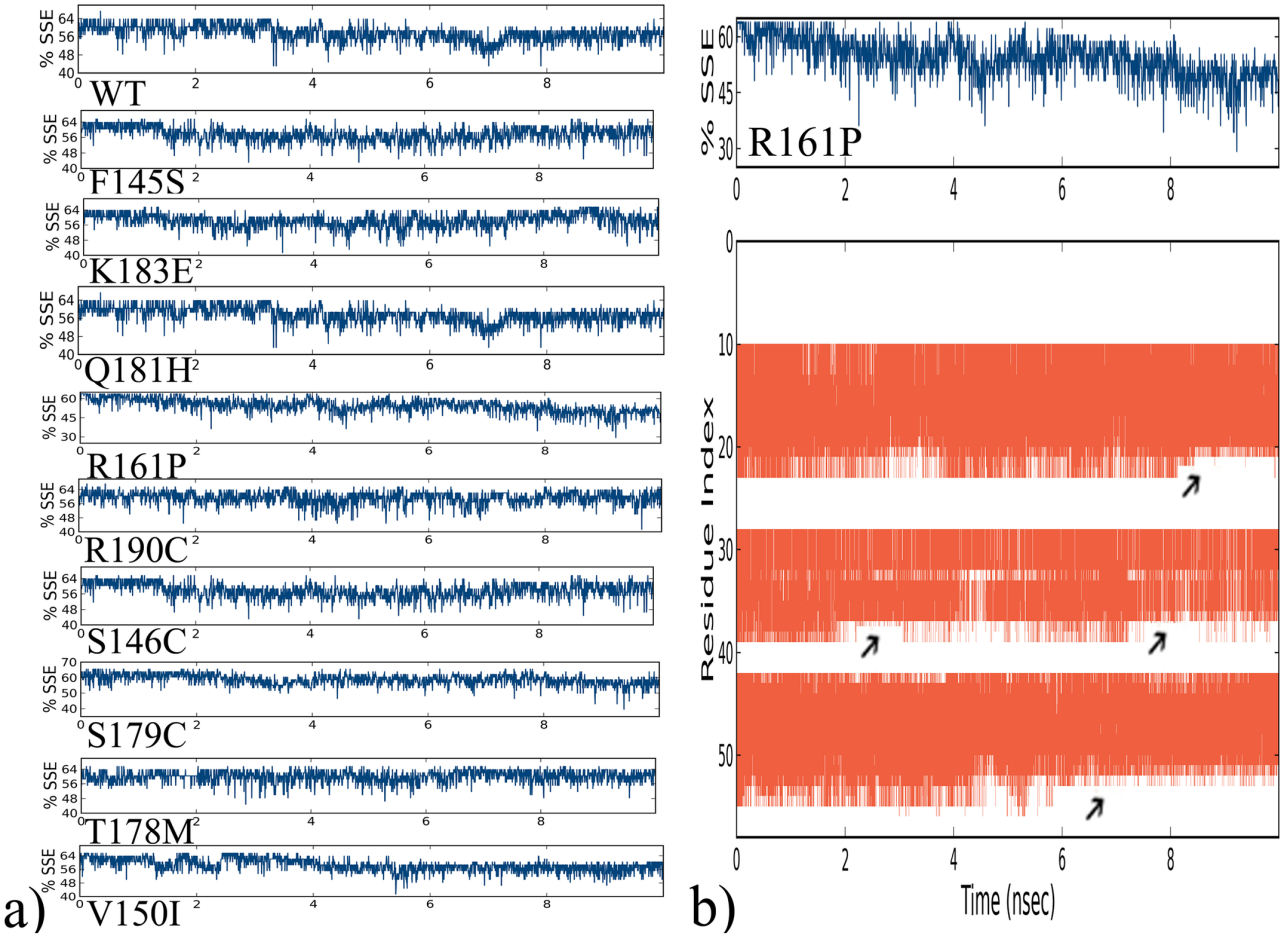
a) Secondary structure element percentage of the native wild type and mutant proteins b) SSE percentage of R161P mutant protein along with its occupancy of helices, strands, turns (orange) and loops (white) along the simulated time of 10ns with reference to the residue index.

**Fig 7:**
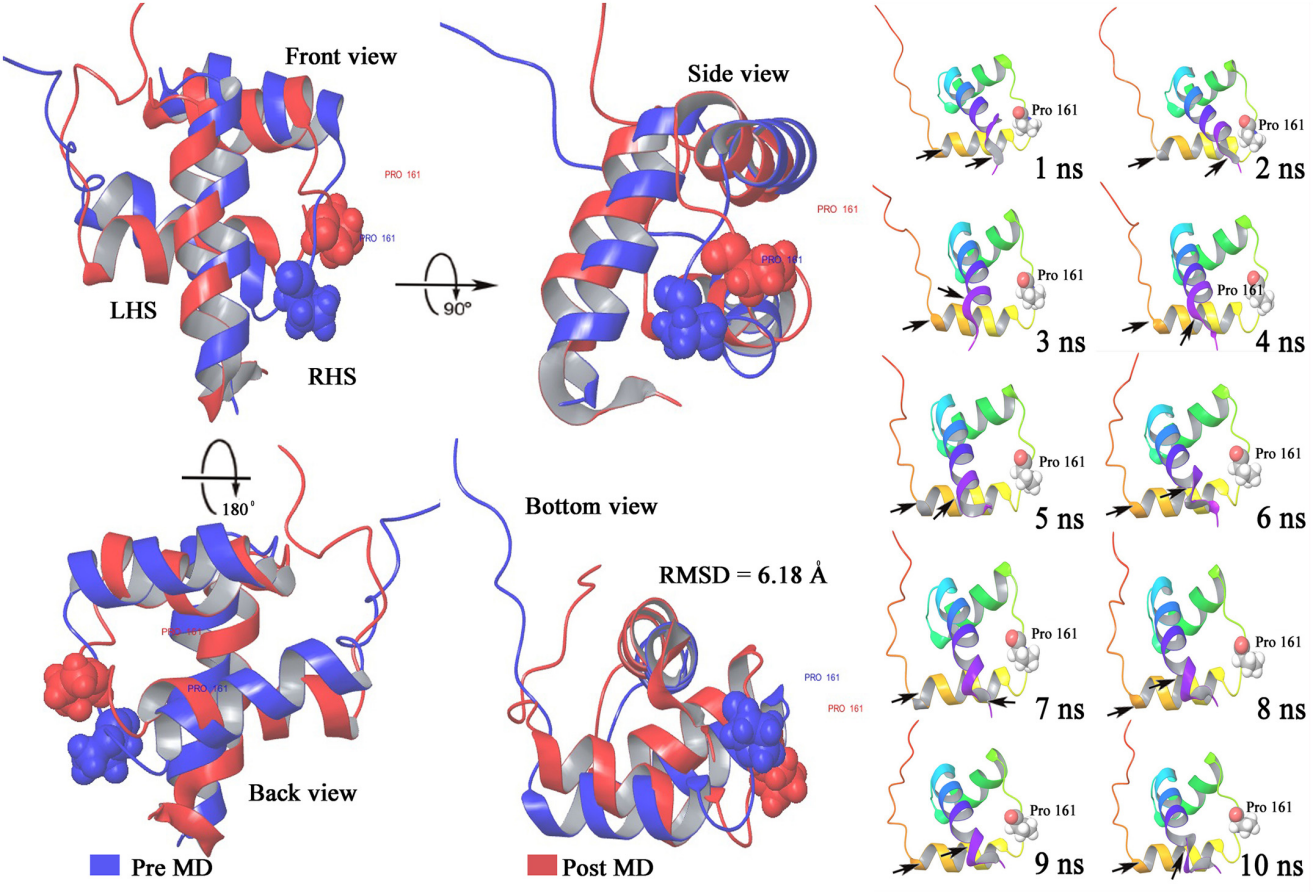
Visualization of the R161P mutant NKX 2–5 protein superimposed of pre (blue) and post (red) MD structures along with snapshot of the simulation trajectory for every 1 nanosecond timescale.

When the secondary structure elements (SSE) contributing to the overall protein stability were analyzed, it was observed that all the proteins were maintaining an average of around 64% SSE, mostly composed of helices rather than strands and loops, with an exception of R161P mutation. When we further investigated why R161P was showing less SSE percentage comparatively, it was revealed that residues present at 20, 40 and 50 series of position (Fig 6) in the wild type were found to be converting from strands to loops leading to the loss of SSE elements and probably causing damage to its overall stability and conformational status (Fig 7).

## 3. Materials and Methods

### 3.1 Selection of SNPs for *in silico* analysis

The dbSNP was used to retrieve SNPs reported in NKX 2–5 gene. (http://www.ncbi.nlm.nih.gov/snp, access date: June 30 2014) [33]. We have selected the nsSNPs s for further analysis.

### 3.2 Prediction of deleterious ns SNPs by *In silico* tools

Many computational biology tools are available that are used to determine whether an nsSNP is expected to be neutral or pathogenic, but we have utilized the most widely accepted computational algorithms, namely Sorting intolerant from tolerant (SIFT) and Polymorphism phenotyping (Polyphen). First, pathogenic nsSNPs in the NKX2-5 gene were identified using these tools and, then a MDS analysis was performed to investigate the possible effects that amino acid variants may have on NKX2-5 protein structure. These methods rely on either sequence information or structural information, or both, to predict the functional impact of SNPs. The methods used in this study were also the same as described in recent publications using default parameters [10].

### 3.3 Sorting tolerant from intolerant

*3*.*3*.*1 SIFT* (http://sift.jcvi.org/www/SIFT_dbSNP.html, access date: June 30 2014) is a multistep algorithm that predicts whether an amino acid substitution would affect protein function or not, by filtering out the mutations based on tolerance score [34]. SNP data was retrieved from dbSNP as rs IDs, then submitted to the SIFT server for analysis. The SIFT server reports the results as prediction scores between 0 and 1. A score within a range of 0–0.05 is considered to be deleterious or pathogenic, whereas scores above 0.05 to 1 are considered to be neutral or non-pathogenic.

*3*.*3*.*2 Polyphen 2*.*0* (http://genetics.bwh.harvard.edu/pph2/dbsearch.shtml; access date July 2 2014) is also a high quality sequence alignment pipeline that uses a combination of sequence and structure based attributes where the effect of mutation is predicted by a native Bayesian classifier [35]. This program has been trained on two pairs of existing data sets, one was the HumDiv and other was Hum var present in the UnitProtKB database. The queries were submitted to the polyphen2 server in the form of dbSNP IDs.The output levels would be appraised qualitatively as benign, possibly damaging(less confident prediction) and probably damaging (more confident prediction) based on pairs of false positive rate (FPR) threshold, which is an added advantage with regards to prediction accuracy. If the Polyphen score is greater than or equals 0.5, it can be classified as deleterious, and if the score is less than 0.5 it can be regarded as benign or tolerated.

*3*.*3*.*3 I-mutant 2*.*0* [36] (http://gpcr2.biocomp.unibo.it/cgi/predictors/I-Mutant3.0/I-Mutant3.0.cgi) is a support vector machine (SVM) based server capable of doing automatic prediction of protein stability changes arising from a single point mutation We used sequence based version of I-mutant 2.0 (access date July 2 2014) and the protein stability change was predicted as increase or decrease for NKX 2–5 protein sequences retrieved from NCBI.

*3*.*3*.*4 PANTHER* [37] (Protein Analysis through Evolutionary Relationships) is a database which uses Hidden Markov Model (HMD) based statistical modeling and multiple sequence alignments to perform evolutionary analysis of nsSNPs. We submitted our queries as protein sequences (access date July 2 2014) and the PANTHER calculates the substitution position-specific evolutionary conservation score (subPSEC) based on an alignment of evolutionary related proteins. PANTHER subPSEC score ranges from 0 to about -10. Protein sequences having a subPSEC score ≤ -3 are considered as most likely to be deleterious.

### 3.4 Modeling of the mutant protein structure

The three-dimensional structure of the protein is crucial in order to study its functionality, especially when trying to understand the effect of SNP’s on its overall structure and function. We used the rcsb.org to identify the protein coded by NKX 2.5 (PDB ID 3RKQ), which is a 58 amino acids in length starting from the residue at position 138 and ending at 194. Even though there were about 49 reported mutations (Table 4) in NKX 2.5 gene, we have studied nine mutation effects due to lack of the 3D structural information of this protein in comparison of the wild type protein. The modeling of the mutated protein structures have been performed using the “mutate a residue” tool in the Schrödinger maestro v9.6 visualization program [38] using the wild type available 3D structure as a reference.

### 3.5 MD simulations in water

"Desmond v3.6 Package" [39–40] was used to run the molecular dynamic simulations. Predefined TIP3P water model [41] was used to simulate water molecules. Orthorhombic periodic boundary conditions were set up to specify the shape and size of the repeating unit buffered at 10 Å distances. In order to neutralize the system electrically, appropriate counter NA+/Cl- ions were added to balance the system charge and were placed randomly in the solvated system. After building the solvated system, we have performed minimization and relaxation of the protein/protein-ligand complex under NPT ensemble using default protocol of Desmond as followed elsewhere [42–43]; which includes a total of 9 stages among which there are 2 minimization and 4 short simulations (equilibration phase) steps are involved before starting the actual production time.

3.5.1 Summary of Desmond’s MD simulation stages:

Stage 1—task (recognizing the simulation setup parameters)
Stage 2—minimize, Minimization with restraints on solute
Stage 3—minimize, Minimization without any restraints
Stage 4—simulate, Berendsen NVT, T = 10 K, small time steps, and restraints on solute heavy atoms.
Stage 5 –simulate, Berendsen NPT, T = 10 K, and restraints on solute heavy atoms
Stage 6—solvate_pocket
Stage 7—simulate, Berendsen NPT and restraints on solute heavy atoms
Stage 8—simulate, Berendsen NPT and no restraints
Stage 9—simulate (production time)

Molecular dynamic simulations were carried out with the periodic boundary conditions in the NPT ensemble using OPLS 2005 force field parameters [44–45]. The temperature and pressure were kept at 300 K and 1 atmospheric pressure respectively using Nose-Hoover temperature coupling and isotropic scaling [46], the operation was followed by running the 10ns NPT production simulation each and saving the configurations thus obtained at 5ps intervals.

### 3.6 Analysis of molecular dynamics trajectory

The MDS trajectory files were analyzed by using simulation quality analysis (SQA), simulation event analysis (SEA) along with simulation interaction diagram (SID) programs available with the Desmond module for calculating the Energies, root-mean-square deviation (RMSD), root-mean square fluctuation (RMSF). Total intra molecular hydrogen bonds, Radius of Gyration along with secondary structure elements (SSE) of the protein contributing to the structural stability. SQA is a useful parameter to qualitatively validate the system stability throughout the simulated length of chemical time for the given temperature, pressure and volume of the total simulation box. Whereas, SEA is used towards analyzing each frame of simulated trajectory output and SID has been employed especially towards estimating the total SSE change in the protein structure during the simulation time.

## 4. Conclusions

The present study offers an insight into the genotype–phenotype association of deleterious nSNPs associated with NKX 2–5 gene. Our study identified nine pathogenic SNPs: rs72554027 (F145S), rs397516909 (S146W), rs201582515 (V150I), rs137852685 (R161P), rs104893900 (T178M), rs3729938 (S179C), rs72554028 (Q181H) rs137852686 (K183E) and rs104893906 (R190C) in NKX2-5. Furthermore, the MDS analysis revealed their respective major consequences on the native homeobox-containing transcription factor, which is encoded by NKX 2–5 gene. RMSD, RMSF, energy parameters, intra molecular H-Bonds, Radius of Gyration and secondary structure element plots revealed their plausible malfunctioning mechanism via their structural destabilization. Compared to the wild type, all the selected mutations were altering the structural behavior of the mutated protein, however R161P (rs137852685) predicted to cause the most damage to the protein’s structural features followed by rs104893906 (R190C). Overall, the present computational approach will provide a comprehensive view on destabilizing mechanisms of homeobox-containing transcription factor SNPs in NKX2-5. The knowledge thus acquired through this present study is expected to help in prioritizing the important nsSNPs to be selected for further wet lab evaluations, and is of high value especially when designing huge population based genotyping studies.

## Supporting Information

S1 Table Summary of nsSNPs Prediction results that were analyzed by four computational methods SIFT, PolyPhen, I-mutant and PANTHER. AA-Amino Acid; NP-No prediction; SIFT Prediction score: Deleterious (≤ 0.05); Tolerated (≥.0.05); PolyPhen Prediction Score: Damaging (≤1.5); Benign (≥1.5); I-Mutant 2.0 Prediction score: Decrease stability (DDG < 0); Increase stability (DDG > 0); PANTHER subPSEC score: Deleterious (> -3); Tolerated (< -3).
(DOC)
