# Supplementary material for: A Comprehensive In Silico Analysis on the Structural and Functional Impact of SNPs in the Congenital Heart Defects Associated with NKX2-5 Gene—A Molecular Dynamic Simulation Approach
Source: PLoS One. 2016 May 6;11(5):e0153999. doi: 10.1371/journal.pone.0153999 (PMC4859487; doi:10.1371/journal.pone.0153999)
Supplement: S1 Table — AA-Amino Acid; NP-No prediction; SIFT Prediction score: Deleterious (≤ 0.05); Tolerated (≥.0.05); PolyPhen Prediction Score: Damaging (≤1.5); Benign (≥1.5); I-Mutant 2.0 Prediction score: Decrease stability (DDG < 0); Increase stability (DDG > 0); PANTHER subPSEC score: Deleterious (> -3); Tolerated (< -3). (DOC) [file pone.0153999.s001.doc]

**S1 Table . Summary of nsSNPs Prediction results that were analyzed by four computational methods SIFT, PolyPhen, I-mutant and PANTHER**

**AA-Amino Acid; NP-No prediction; SIFT Prediction score: Deleterious (≤ 0.05); Tolerated (≥.0.05); PolyPhen Prediction Score: Damaging (≤1.5); Benign (≥1.5); I-Mutant 2.0 Prediction score: Decrease stability**

| **Gene- NKX2-5** |  |  |  | **SIFT** | | **PolyPhen** | | **I-Mutant 2.0** | | **PANTHER** | |  |
| --- | --- | --- | --- | --- | --- | --- | --- | --- | --- | --- | --- | --- |
|  | **SNP IDs** | **Allele** | **AA**  **change** | **Tolerance**  **Index** | **Prediction** | **PSIC** | **Prediction** | **DDG** | **Prediction** | **subPSEC** | **Prediction** |  |
|  | rs376426882 | C/G | R322P | 0.00 | Deleterious | 0.859 | Damaging | -1.05 | Decrease stability | -0.88461 | Tolerated |  |
|  | rs201249977 | C/A | V315L | 0.33 | Tolerated | 0.003 | Damaging | -0.62 | Decrease stability | -0.68847 | Tolerated |  |
|  | rs201249977 | C/T | V315M | 0.09 | Tolerated | 0.037 | Damaging | -2.01 | Decrease stability | -1.41222 | Tolerated |  |
|  | rs200152391 | C/T | G314E | 0.02 | Deleterious | 0.972 | Damaging | 0.33 | Increase stability | -0.87072 | Tolerated |  |
|  | rs200152391 | C/G | G314A | 0.25 | Tolerated | 0.91 | Damaging | -0.76 | Decrease stability | -0.88146 | Tolerated |  |
|  | rs142368156 | C/T | S311N | 0.17 | Tolerated | 0.00 | Damaging | -0.14 | Decrease stability | -1.66801 | Tolerated |  |
|  | rs371380388 | G/T | A302E | 0.92 | Tolerated | 0.031 | Damaging | -0.61 | Decrease stability | -0.96511 | Tolerated |  |
|  | rs137852683 | T/C | D299G | 0.31 | Tolerated | 0.018 | Damaging | -2.4 | Decrease stability | -1.08194 | Tolerated |  |
|  | rs549406766 | C/T | G298E | 0.05 | Deleterious | 0.972 | Damaging | -0.35 | Decrease stability | -0.38903 | Tolerated |  |
|  | rs569535312 | C/A | 0.23899 | 0.16 | Tolerated | 0.884 | Damaging | -1.34 | Decrease stability | -1.84179 | Tolerated |  |
|  | rs373421818 | C/T | G296D | 0.46 | Tolerated | 0.028 | Damaging | -1.11 | Decrease stability | -0.87485 | Tolerated |  |
|  | rs150581386 | G/C | F295L | 1.00 | Tolerated | 0.483 | Damaging | -0.88 | Decrease stability | -0.45365 | Tolerated |  |
|  | rs538010963 | G/C | F292L | 0.15 | Tolerated | 0.483 | Damaging | -0.63 | Decrease stability | -0.54522 | Tolerated |  |
|  | rs375086983 | G/T | P283Q | 0.16 | Tolerated | 0.061 | Damaging | -0.47 | Decrease stability | NP | - |  |
|  | rs571382279 | A/C | S279A | 1.00 | Tolerated | 0.00 | Damaging | 1.63 | Increase stability | -1.16022 | Tolerated |  |
|  | rs368366482 | G/T | P275T | 0.01 | Deleterious | 0.908 | Damaging | -2.47 | Decrease stability | -1.22005 | Tolerated |  |
|  | rs553883993 | T/A | Y259F | 0.05 | Deleterious | 0.636 | Damaging | -0.67 | Decrease stability | -1.97809 | Tolerated |  |
|  | rs387906776 | G/C | P257A | 0.71 | Tolerated | 0.811 | Damaging | -1.69 | Decrease stability | -0.84327 | Tolerated |  |
|  | rs397515399 | G/T | P236H | 0.04 | Deleterious | 0.452 | Damaging | -1.85 | Decrease stability | -2.40029 | Tolerated |  |
|  | rs104893902 | G/A | A219V | 0.00 | Deleterious | 0.939 | Damaging | 0.98 | Increase stability | -3.13961 | Deleterious |  |
|  | rs104893905 | G/A | R216C | 0.00 | Deleterious | 0.973 | Damaging | -0.29 | Decrease stability | -6.87255 | Deleterious |  |
|  | rs372282873 | G/C | P212R | 0.00 | Deleterious | 0.965 | Damaging | -0.49 | Decrease stability | NP | _ |  |
|  | rs3729754 | G/A | P211L | 0.31 | Tolerated | 0.982 | Damaging | -0.33 | Decrease stability | NP | _ |  |
|  | rs104893906 | G/A | R190C | 0.00 | Deleterious | 0.941 | Damaging | -0.51 | Decrease stability | -12.93433 | Deleterious |  |
|  | rs137852686 | T/C | K183E | 0.00 | Deleterious | 0.663 | Damaging | 0.28 | Increase stability | -6.18997 | Deleterious |  |
|  | rs72554028 | C/A | Q181H | 0.00 | Deleterious | 0.986 | Damaging | -0.94 | Decrease stability | -5.82919 | Deleterious |  |
|  | rs3729938 | G/C | S179C | 0.01 | Deleterious | 0.993 | Damaging | -1.24 | Decrease stability | -5.39192 | Deleterious |  |
|  | rs104893900 | G/A | T178M | 0.00 | Deleterious | 0.996 | Damaging | -0.86 | Decrease stability | -6.75592 | Deleterious |  |
|  | rs137852685 | C/G | R161P | 0.00 | Deleterious | 0.953 | Damaging | -0.55 | Decrease stability | -5.62458 | Deleterious |  |
|  | rs201582515 | C/T | V150I | 0.00 | Deleterious | 0.935 | Damaging | 0.72 | Increase stability | -3.45988 | Deleterious |  |
|  | rs397516909 | G/C | S146W | 0.00 | Deleterious | 0.996 | Damaging | 0.6 | Increase stability | -7.94671 | Deleterious |  |
|  | rs72554027 | A/G | F145S | 0.00 | Deleterious | 0.976 | Damaging | -2.16 | Decrease stability | -6.81332 | Deleterious |  |
|  | rs369025518 | G/A | A119V | 0.28 | Tolerated | 0.001 | Damaging | -0.48 | Decrease stability | -2.38303 | Tolerated |  |
|  | rs137852684 | C?A | A119S | 1.0 | Tolerated | 0.021 | Damaging | -0.93 | Decrease stability | -1.52288 | Tolerated |  |
|  | rs112167223 | A/C | L116R | 0.05 | Deleterious | 0.137 | Damaging | -1.86 | Decrease stability | -1.38998 | Tolerated |  |
|  | rs529610517 | G/A | A115V | 0.29 | Tolerated | 0.022 | Damaging | -0.18 | Decrease stability | -2.38927 | Tolerated |  |
|  | rs200039950 | C/T | R143Q | NP | - | NP | - | -0.92 | Decrease stability | NP | - |  |
|  | rs534163213 | G/A | A112V | 0.15 | Tolerated | 0.034 | Damaging | -0.54 | Decrease stability | NP | - |  |
|  | rs550046293 | G/C | P100A | 0.76 | Tolerated | 0.01 | Damaging | -1.21 | Decrease stability | -2.15042 | Tolerated |  |
|  | rs373807012 | A/G | F86S | 0.51 | Tolerated | 0.354 | Damaging | -0.46 | Decrease stability | NP | - |  |
|  | rs150813574 | A/T | C82S | 0.53 | Tolerated | 0.879 | Damaging | -1.63 | Decrease stability | NP | - |  |
|  | rs201362118 | C/T | G74D | 0.63 | Tolerated | 0.992 | Damaging | -1.79 | Decrease stability | NP | - |  |
|  | rs530270916 | G/A | A63V | 0.28 | Tolerated | 0.939 | Damaging | 1 | Increase stability | NP | - |  |
|  | rs387906775 | G/C | P59A | 0.81 | Tolerated | 0.956 | Damaging | -0.79 | Decrease stability | NP | - |  |
|  | rs549161381 | C/G | A57P | 0.2 | Tolerated | 0.001 | Damaging | -0.43 | Decrease stability | NP | - |  |
|  | rs567939950 | C/T | A55T | 0.54 | Tolerated | 0.124 | Damaging | -1.38 | Decrease stability | NP | - |  |
|  | rs113818864 | C/G | A42P | 0.25 | Tolerated | 0.981 | Damaging | 0.42 | Decrease stability | NP | - |  |
|  | rs552617433 | C/T | E32K | 0.37 | Tolerated | 0.023 | Damaging | -0.35 | Decrease stability | NP | - |  |
|  | rs28936670 | G/A | R25C | 0.01 | Deleterious | 0.973 | Damaging | 0.05 | Increase stability | NP | - |  |
|  | rs201442000 | T/C | Q22R | 0.03 | Deleterious | 0.775 | Damaging | -0.71 | Decrease stability | NP | - |  |
|  | rs104893904 | C/G | E21Q | 0.04 | Deleterious | 0.932 | Damaging | 0.42 | Increase stability | NP | - |  |
|  | rs17052019 | T/G | D16A | 0.00 | Deleterious | 0.968 | Damaging | 0 | Decrease stability | NP | - |  |
|  | rs387906773 | T/A | K15I | 0.00 | Deleterious | 0.979 | Damaging | 0.46 | Decrease stability | NP | - |  |

**(DDG < 0); Increase stability (DDG > 0); PANTHER subPSEC score: Deleterious (> -3); Tolerated (< -3).**
